# 

**Published:** 1905-03-18

**Authors:** 


### NOTICE TO CORRESPONDENTS.

All MSS., Letters, Books for Review, and other matters intended for the Editor should be addressed The Editor, "The Hospital," The Hospital Buildings, 28 & 29 Southampton Street, Strand, W.C.

The Editor cannot undertake to return rejected MS., even when accompanied by stamped directed envelope.

### Notice to Advertisers and Subscribers.

All Advertisements, Orders for Copies of The Hospital, and Business Communications should be addressed to **THE MANAGER (Not to the Editor),** The Hospital, 28 & 29 Southampton Street, Strand, London, W.C.

The Cost of Subscription, post free, is as follows:—Per week, 3½d.; per quarter, 3s. 3d.; per half-year, 6s. 6d.; per annum, 13s. Foreign and Colonial:—Per annum, 19s. 6d., payable, in all cases, in advance.

**NOTICE.—Vol. XXXVI. of "The Hospital" (April to September 1904), in handsome cloth binding, is now on sale at the office of this Journal, price 10s. 6d. Cases for binding the Numbers contained in this Volume 2s. Index to Vol. XXXVI., 3½d., post free.**

**The Monthly part for March is now ready. Price 1s., by post, 1s. 3d.**

### BOOKS RECEIVED.

Adlard and Son.

"In Confidence to Boys." By H. Bisseker, M.A.

P. S. King and Son.

"Infantile Mortality and Infants' Milk Depots." By G. F. McCleary, M.D., D.P.H.

J. Wright & Co., Bristol.

"Medical Annual, 1905."

Rivington's.

"Health at School." Clement Dukes, M.D.

### MEDICAL APPOINTMENTS.

**Augustus William Dalby**, L.R.C.P.&S.Edin., reappointed Medical Officer of Health of the Frome (Somerset) Rural District. **Henry King Dawson**, M.D., B.S.Durh. Public Vaccinator of the Epsom and Ashtead Parishes of the Epsom Union. **C. Farrant**, M.R.C.S., L.R.C.P.Lond., Certifying Surgeon under the Factory and Workshop Act for the Taunton District of the county of Somerset. **John W. Geddes**, M.B., C.M.Edin., Medical Superintendent of the Middlesbrough County Borough Asylum, vice Dr. G. S. Pope. **Matthew Richard Gooding**, M.R.C.S., L.R.C.P.Lond., reappointed Medical Officer for the Bideford (Devon) Urban District Council. **Alfred Pearce Gould**, M.S., F.R.C.S., Consulting Surgeon to the Bolingbroke Hospital. **J. T. C. Nash**, M.D.Edin., Medical Officer to the Southend-on-Sea Education Authority. **Philip Nelson**, M.D.Vict., M.D.Liverpool, Honorary Assistant Physician and Laryngologist to the Liverpool Chest Hospital. **S. Partridge**, M.B.Edin., Certifying Surgeon under the Factory and Workshop Act for the Wednesbury District of the County of Stafford. **James Pearse**, M.D., C.M.Edin., reappointed Medical Officer of Health for Trowbridge (Wilts) Urban District Council. **O. H. Rogerson**, L.S.A., Certifying Surgeon under the Factory and Workshop Act for the Osmotherley District of the County of York. **Charters J. Symonds**, M.S., F.R.C.S., Consulting Surgeon to the Bolingbroke Hospital. **E. S. Worrall**, M.R.C.S.Eng., L.R.C.P.Lond., Medical Officer in Charge of the New Electro-therapeutic Department, University College Hospital.

**"The Hospital" Institutional Library.**

**"Hospitals and Asylums of the World." 4 Vols., with a Portfolio of Plans, complete £12 12s.**
**"Burdett's Hospitals and Charities, 1904." 5s. net.**
**"Burdett's Official Nursing Directory." 8s. net.**
**"Cottage Hospitals, General, Fever, and Convalescent." 10s. 6d.**
**"Uniform System of Accounts for Hospitals and Public Institutions." New Edition. 4s. net.**
**"Hospital Expenditure: The Commissariat." 2s. 6d.**
**"A Legal Handbook for the Use of Hospital Authorities." 2s. 6d.**
**"The Cottage Hospital Case Book or Register of Patients." 10s. and 12s. 6d.**
**All these are published by the SCIENTIFIC PRESS, Ltd., and may be obtained through any bookseller, or direct from the publishers, 28 and 29 Southampton Street, Strand, London, W.C.**

Post free.

**HOW TO BECOME A CERTIFIED MIDWIFE.** By E. L. C. Appel, M.B., B.S., B.Sc. Lond. - - - - - - 1/- net 1/1

**PRELIMINARY LECTURES ON MIDWIFERY.** By Margaret M. Caley, late Matron, Fulham Midwifery Training School. 1/6 net 1/8

**DISTRICT MIDWIFERY CASE-BOOK.** Compiled by Margaret Caley, late Matron, Fulham Midwifery Training School. Suitable size for the Pocket - - - - - 6d. net. 7d.

**THE MIDWIVES' POCKET-BOOK.** By Honnor Morten - - - - - - - - 1/-

**ON PREPARATION FOR OPERATION IN PRIVATE HOUSES.** By Stanmore Bishop, F.R.C.S. - - - - - - - 6d.

**PRACTICAL HINTS ON DISTRICT NURSING.** By Miss Amy Hughes - - 1/-

**THE "SIMPLEX" DISTRICT NURSES' CASE-BOOK.** Containing space for 50 cases, ruled and printed, bound in cloth boards, 6d. net 7d.

Post free.

**A COMPLETE HANDBOOK OF MIDWIFERY.** By J. K. Watson, M.D. Edin., Author of "A Handbook for Nurses." Profusely illustrated with 143 Diagrams 6/- net 6/4

**A PRACTICAL HANDBOOK OF MIDWIFERY.** By Francis W. Haultain, M.D. Profusely illustrated, Tables, &c. Handsomely bound in leather - - - - 6/- net 6/3

**NURSING NOTES ON MIDWIFERY AND GYNÆCOLOGY.** By Sister Ross, Rotunda Hospital, Dublin - - 2/- net 2/1

**ART OF FEEDING THE INVALID.** By a Medical Practitioner and a Lady Professor of Cookery. With Special Chapter on Diet for Consumptives - - - - 1/6

**THE POCKET CASE BOOK FOR DISTRICT AND PRIVATE NURSES.** By a Physician - - - - - - - - 1/-

**POCKET-BOOK OF TEMPERATURE CHARTS.** Containing 17 Twelve-hour and 8 Four-hour Charts, perforated so that each Chart may be removed after use - 6d. net 7d.

**TEMPERATURE CHARTS, MORNING AND EVENING, and FOUR-HOUR CHARTS.** Carriage paid.

| | | | | |
|---|---|---|---|---|
| Size 10 inches by 8 inches | 25 | 1/- | 250 | 6/6 |
| | 50 | 1/6 | 500 | 12/- |
| | 100 | 2/9 | 1,000 | 23/- |

THE SCIENTIFIC PRESS, LTD., 28 & 29 Southampton Street, Strand, London, W.C.

### Nurses, You Need Cards.

EVERY HOSPITAL and PRIVATE NURSE should obtain a supply of Professional Cards. In order to meet the demand in this direction we have started a Department for the production of Personal Cards at greatly Reduced Prices.

**For 2/- we will send you, Post Free, 100 Best Ivory Cards,**

Printed in either of the Types shown below, including Name and Address and Professional Distinctions, and neatly packed in Cardboard Box.

No. 1. — STYLES OF TYPE. — No. 2.

*Nurse Evetts,*

*Certificated Medical & Surgical Monthly Nurse.*

Nurse Shergold,

Certificated Q.C.H., L.O.S.

Name and particulars should be plainly written. Any other Type specially required by Customer will be matched or followed as closely as possible.

Address, THE MANAGER—

The SCIENTIFIC PRESS, LTD., 28 & 29 Southampton St., Strand, London, W.C.

